# Editorial: nTMS, Connectivity and Neuromodulation in Brain Tumor Patients

**DOI:** 10.3389/fneur.2022.885773

**Published:** 2022-04-06

**Authors:** Giovanni Raffa, Thomas Picht, András Büki, Antonino Germanò

**Affiliations:** ^1^Division of Neurosurgery, BIOMORF Department, University of Messina, Messina, Italy; ^2^Department of Neurosurgery, Charité Universitätsmedizin Berlin, Berlin, Germany; ^3^Department of Neurosurgery, Faculty of Medicine and Health, Örebro University, Örebro, Sweden

**Keywords:** brain connectivity, brain mapping, brain tumors, neuromodulation, nTMS, preoperative planning, tractography

Surgery of brain tumors still represents a challenge for neurosurgeons, especially if these lesions are located close to or eventually infiltrate functionally-eloquent brain networks (1). The goal of surgery is to achieve the maximal safe resection of the neoplastic tissue while preserving the surrounding functionally-relevant brain areas (e.g., sensorimotor, language, visual networks) (2). Such a goal can be achieved by combining technological advancements in surgical techniques with knowledge of the brain's anatomic and functional organization.

In recent years we have witnessed a paradigm shift in knowledge about how the brain works: the historical localizationist interpretation of brain functional organization has been recently replaced by the hodotopical model, in which brain functions do not correspond to fixed anatomical areas but rely on complex cortico-subcortical networks with huge plastic potential (3). Neuroplasticity is responsible for the final connectomic organization of functional brain networks and brain tumor patients usually show huge neuroplasticity phenomena. Disclosing these neuroplasticity phenomena and investigating connectomics underlying the brain functional organization in brain tumor patients is essential to plan and achieve the maximal safe resection of the tumor that is associated with improved outcome and prolonged survival (4). Several electrophysiological and neuroimaging techniques can be used before and during surgery to map the spatial relationship between the tumor and adjacent eloquent networks that must be preserved during surgery. Among these, transcranial magnetic stimulation (TMS), transcranial electrical stimulation (TES), functional MRI (fMRI), magnetoelectroencephalography (MEG), tractography, and finally awake surgery and direct electric stimulation (DES) are the most used. In addition, electrophysiological approaches have been developed to analyze and induce neuroplasticity in these patients before and after surgery, aiming to stimulate brain functional reorganization, making resection safer, and promoting postoperative functional recovery (5, 6).

All these different imaging and neurophysiological advanced techniques, or specific combinations of them, provide a unique and modern *in-vivo* connectomic analysis of the brain functional organization in every single patient. Those analyses enable a personalized strategy for surgical resection and post-operative rehabilitation and neurological recovery, resulting in a better patients' outcome and quality of life.

Nevertheless, the integration of information provided by these modern tools requires a multidisciplinary approach and a strong collaboration between different professionals, including neuroscientists, neurologists, neuroradiologists, neuropsychologists, and neurosurgeons with strong expertise in the field. A comprehensive review of the technical aspects of such a multidisciplinary approach for connectomics analysis before and during surgery of brain tumors is still lacking. The present Article Collection attempted to provide new insights into novel multidisciplinary approaches to perform advanced connectomics analysis of brain functional organization through the description of the modern pre- and intraoperative electrophysiological and neuroimaging techniques to plan and achieve the maximal safe resection of brain tumors, as well as the illustration of novel neuromodulation approaches based on advanced brain stimulation techniques used to disclose neuroplasticity phenomena, and promote neurological reorganization before surgery.

Among preoperative neurophysiological tools for connectomic analysis, navigated TMS (nTMS) is one of the most widely diffused in Neurosurgical Departments all over the world. Several studies demonstrated nTMS provides a reliable mapping of the motor cortex and language cortical areas, thus allowing neurosurgeons to plan the safest surgical strategy to remove brain tumors close to these networks (7–13). In the present Article Collection, Umana et al. performed an updated systematic review and meta-analysis of literature regarding the use of nTMS mapping before brain tumor surgery: the authors confirmed that nTMS is a safe technique that, in association with DES, improves brain mapping and the extent of tumor resection, thus favoring a better patients' postoperative outcome. Similarly, a study by Haddad et al. summarized the current evidence supporting the efficacy of preoperative nTMS mapping in improving the surgical planning, tumor resection, and postoperative outcome in brain tumor patients. However, the nTMS motor mapping is useful not only to plan tumor surgical resection but also to define a safer radiotherapy plan: Dzierma et al. demonstrated that, in patients with motor-eloquent brain metastases, the inclusion of nTMS motor information into the radiotherapy treatment planning is possible with a straightforward workflow and can achieve reduced doses to the nTMS-defined motor area without compromising coverage of the planning target volume. Finally, three different studies demonstrated that the nTMS motor mapping can also be used to reveal preoperative alterations of the cortical excitability in patients with motor-eloquent brain tumors that could be related to and predict the occurrence of motor deficits (Neville et al.; Machetanz et al.; Rizzo et al.).

However, the nTMS cortical mapping of complex functions, including language, is more difficult and reliable than mapping the motor cortex. Weiss Lucas et al. here proposed a novel robust and reliable picture naming tool, optimized for clinical use, to map and monitor language functions in brain tumor patients during preoperative nTMS mapping and awake surgery. On the other hand, Hazem et al. in their study proposed a novel target for the nTMS preoperative mapping of language cortical areas: in particular, they demonstrated that the posterior middle frontal gyrus, including the area 55b, is an important integration cortical hub for both dorsal and ventral streams of language and can be successfully used as a target for the nTMS language cortical mapping.

A few data are currently available about the possibility to use nTMS for mapping cortical areas involved in brain functions other than language. Raffa et al. submitted a paper demonstrating nTMS can be used also for mapping the cortical areas of the right parietal lobe involved in visuospatial abilities. The authors reported that the nTMS mapping of these areas, in combination with the tractography of the superior longitudinal fascicle (SLF), can be used to plan and achieve the maximal safe resection of brain tumors located in the right parietal lobe, without inducing a worsening of patients' visuospatial abilities.

All the previous studies refer to the nTMS cortical mapping in adult brain tumor patients. However, in the previous literature, only anecdotic reports analyzed the feasibility of the nTMS mapping in children. Narayana et al. performed a study demonstrating that TMS is a safe, reliable, and effective tool to map eloquent cortices also in a series of 36 young children (3 years old or younger). In this study the TMS mapping improved understanding the risk-benefit ratio prior to surgery and facilitated surgical planning aimed at preserving motor, speech, and language functions.

Nevertheless, the nTMS mapping enables only the visualization of the cortical areas of functional networks (motor/language/visuospatial) that must be preserved during the resection of the tumor. Therefore, the data from nTMS cortical mapping must be combined with the tractography of the respective subcortical pathways, aiming at the preoperative identification of the entire functional network (14–16). Rosenstock et al. demonstrated that the nTMS motor mapping can be successfully combined with the tractography of the corticospinal tract to stratify patients with motor-eloquent tumors before surgery, distinguishing between patients with a high vs. low risk of developing new postoperative motor deficits. Such a stratification is particularly useful during surgery, by helping neurosurgeons to interpret ambiguous intraoperative monitoring phenomena (such as irreversible MEP amplitude decrease ≤50%) and to adjust the subcortical stimulation intensity. In another study, Fekonja et al. showed that the analysis of tractography measurements along the corticospinal tract (regardless of using a deterministic or probabilistic approach) is useful to stratify patients with motor-eloquent gliomas by disclosing tumor-induced changes in the structural integrity of the tract in the affected hemisphere.

A similar preoperative patient stratification can be achieved also in cases of language-eloquent tumors by performing the tractography of fascicles involved in the language network. Ius et al. in their study, computed the tractography of the superior longitudinal fascicle (SLF) and inferior fronto-occipital fascicle (IFOF) to stratify patients with language-eloquent low-grade gliomas. In particular, the authors demonstrated that the comparison of quantitative parameters resulting from the DTI tractography between the tumoral vs. the healthy hemisphere is useful to assess the risk of post-operative transient language impairment in these patients. Moreover, Di Cristofori et al. performed a review to analyze the current literature evidence about the possible role of the preoperative assessment of the asymmetry of the arcuate fascicle (AF) by DTI tractography for the preoperative risk assessment of patients undergoing surgical resection of gliomas. They also reported the usefulness of the analysis of AF asymmetry in the health vs. affected hemisphere to predict recovery from aphasia and reorganization of the language brain network even after surgical damage. Finally, Zoli et al. reported an interesting study demonstrating that DTI tractography of the AF is useful not only for planning the safest surgical strategy for tumor resection but also to analyze some along-tract DTI metrics that can provide useful information for differentiating low-grade and high-grade tumors. Another important subcortical white matter tract involved in the language network, the Frontal Aslant Tract (FAT), has been analyzed in the study La Corte et al. The authors performed a systematic review of the literature about the FAT anatomical connectivity and functional roles, thus providing an overview for practical neurosurgical applications in patients with brain tumors: they also eventually suggested the evaluation of FAT integrity by tractography could be useful to plan a safer surgery and to reduce post-operative deficits in patients with language-eloquent brain tumors.

Another important preoperative tool to identify functional networks before brain tumor surgery is fMRI. Nevertheless, some concerns about its accuracy, especially in comparison with nTMS, have been reported in the literature (17). In particular, some criticisms have been raised concerning the accuracy of the motor tasks used for mapping the motor network, especially in patients with brain tumor-related motor deficits (18). Ciavarro et al. performed a very interesting study proposing a novel motor task for a more accurate preoperative localization of the motor cortex in brain tumor patients: the proposed visual-triggered finger movement task (VFMT) resulted to be more reliable than the standard finger-tapping task for the identification of the hand-knob region and showed good correspondence to intraoperative DES. They concluded the VFMT could be very helpful for planning the safest surgical strategy in patients with motor-eloquent tumors.

Recently, neuromodulation by non-invasive brain stimulation (NIBS) has been proposed in the literature as a novel strategy to increase the safety of surgical resection of brain tumors. This strategy aims to promote neuroplasticity and connectivity changes in brain functional networks at surgical risk, thus allowing a “shift” of eloquent structures far away from the tumor. The father of the hodotopical revolution, Duffau, in his Editorial discussed all the potential applications of NIBS to elicit neuroplasticity and facilitate reoperation for low-grade glioma relapse. Moreover, Lang et al. performed a very interesting proof of concept pilot study demonstrating that transcranial direct current stimulation (tDCS) can be used to modify the connectivity of the sensorimotor network in glioma patients. Such a pioneering study confirms the potential usefulness of neuromodulation by NIBS even in the clinical practice, for safer surgical management of brain tumors. Finally, Leao et al. demonstrated that neuromodulation may be used also for the treatment of specific medical conditions, such as tinnitus. The authors reported that low-frequency repetitive TMS to the right dorsolateral prefrontal cortex in patients affected by vestibular schwannomas can result in a significant acute but limited long-term effect on tinnitus.

Apart from preoperative techniques, also intraoperative imaging, neurophysiological and neuropsychological tools play an important role in achieving the safe surgical resection of brain tumors. Barbagallo et al., in their study, demonstrated the importance of a multimodal approach based on different intraoperative imaging techniques (5-ALA, ultrasound, CT scan, etc.) to achieve the maximal safe resection of high-grade gliomas and to improve the patients' postoperative outcome. Giammalva et al. performed a systematic review to assess the role of preoperative connectivity analysis and intraoperative brain mapping to guide the supratotal resection (SpTR) of gliomas, also analyzing the clinical impact of SpTR. They concluded SpTR is related to a longer overall and progression-free survival along with preserving neuro-cognitive functions and quality of life. Herbet et al. reported a perspective article highlighting the possibility to map advanced cognitive functions and behaviors (e.g., multidetermined cognitions such as contextual decision-making or fast learning) during wide-awake surgery. Finally, Sala et al., propose a very interesting opinion study, suggesting novel strategies for the intraoperative assessment of brain connectivity in the anesthetized patient. The aim is to overcome the limitations of the standard neurophysiological mapping and monitoring techniques during asleep surgery and increase the safety of brain tumor resection when awake surgery is not feasible.

In conclusion, this Article Collection includes several remarkable studies summarizing the state-of-the-art of modern preoperative and intraoperative brain stimulation and neuroimaging techniques for performing connectomic analysis and promoting neuroplasticity in brain tumor patients. The scope is to highlight the most novel strategies to enhance knowledge about brain functional organization in brain tumor patients, aiming to improve their surgical treatment and outcome. We believe this Collection will encourage future clinical studies on this fascinating topic and open novel, tailored, safer, and hopefully more effective therapeutic perspectives for patients harboring brain tumors.

## Author Contributions

GR, TP, AB, and AG: conceptualization, collection of the data, writing of the draft, and revision of the final manuscript. All authors are accountable for the content of the work.

## Funding

This editorial and the article collection have been funded by a grant from the European Social Fund (FSE), Call PON Ricerca e Innovazione 2014-2020—AIM Attraction and International Mobility, Activity AIM1839117-3, Line 1, Area SNSI Salute (CUP J44I18000280006), Recipient: GR, BIOMORF Department, University of Messina, Italy. TP acknowledges the support of the Cluster of Excellence *Matters of Activity*. *Image Space Material* funded by the Deutsche Forschungsgemeinschaft (DFG, German Research Foundation) under Germany's Excellence Strategy—EXC 2025−390648296.

## Conflict of Interest

The authors declare that the research was conducted in the absence of any commercial or financial relationships that could be construed as a potential conflict of interest.

## Publisher's Note

All claims expressed in this article are solely those of the authors and do not necessarily represent those of their affiliated organizations, or those of the publisher, the editors and the reviewers. Any product that may be evaluated in this article, or claim that may be made by its manufacturer, is not guaranteed or endorsed by the publisher.
